# The Effectiveness of Nitrate-Mediated Control of the Oil Field Sulfur Cycle Depends on the Toluene Content of the Oil

**DOI:** 10.3389/fmicb.2017.00956

**Published:** 2017-05-31

**Authors:** Navreet Suri, Johanna Voordouw, Gerrit Voordouw

**Affiliations:** Petroleum Microbiology Research Group, Department of Biological Sciences, University of Calgary, CalgaryAB, Canada

**Keywords:** nitrate, nitrate-reducing bacteria, oil fields, toluene, souring control

## Abstract

The injection of nitrate is one of the most commonly used technologies to impact the sulfur cycle in subsurface oil fields. Nitrate injection enhances the activity of nitrate-reducing bacteria, which produce nitrite inhibiting sulfate-reducing bacteria (SRB). Subsequent reduction of nitrate to di-nitrogen (N_2_) alleviates the inhibition of SRB by nitrite. It has been shown for the Medicine Hat Glauconitic C (MHGC) field, that alkylbenzenes especially toluene are important electron donors for the reduction of nitrate to nitrite and N_2_. However, the rate and extent of reduction of nitrate to nitrite and of nitrite to nitrogen have not been studied for multiple oil fields. Samples of light oil (PNG, CPM, and Tundra), light/heavy oil (Gryphon and Obigbo), and of heavy oil (MHGC) were collected from locations around the world. The maximum concentration of nitrate in the aqueous phase, which could be reduced in microcosms inoculated with MHGC produced water, increased with the toluene concentration in the oil phase. PNG, Gryphon, CPM, Obigbo, MHGC, and Tundra oils had 77, 17, 5.9, 4.0, 2.6, and 0.8 mM toluene, respectively. In incubations with 49 ml of aqueous phase and 1 ml of oil these were able to reduce 22.2, 12.3, 7.9, 4.6, 4.0, and 1.4 mM of nitrate, respectively. Nitrate reduced increased to 35 ± 4 mM upon amendment of all these oils with 570 mM toluene prior to incubation. Souring control by nitrate injection requires that the nitrate is directed toward oxidation of sulfide, not toluene. Hence, the success of nitrate injections will be inversely proportional to the toluene content of the oil. Oil composition is therefore an important determinant of the success of nitrate injection to control souring in a particular field.

## Introduction

Sulfide accumulation in oil reservoirs through the reduction of sulfate in injection waters by sulfate-reducing bacteria (SRB) is referred to as souring and is highly undesirable. Addition of nitrate to these injection waters is a commonly used strategy to mitigate the negative impact of sulfide produced in oil fields. Nitrate promotes the growth of sulfide-oxidizing and heterotrophic nitrate-reducing bacteria (soNRB and hNRB). Among these, soNRB oxidize sulfide directly while hNRB exclude SRB growth by competitive utilization of oil organics for nitrate reduction (Sunde and Torsvik, 2005; Youssef et al., 2009; Gieg et al., 2011; Voordouw, 2011). Volatile fatty acids (VFA, a mixture of acetate, butyrate, and propionate) and low molecular weight hydrocarbons such as alkylbenzenes are preferred oil organics for nitrate reduction by hNRB. Incomplete reduction of nitrate to nitrite is a key to the success of this approach to control souring. Nitrite accumulation in oil reservoirs is stimulated at high-temperatures (50–70°C). But nitrite is reduced further to dinitrogen (N_2_) in low-temperature oil fields (below 50°C) in view of the excess of electron donors, which is usually present in oil fields (Reinsel et al., 1996; Agrawal et al., 2012, 2014; Fida et al., 2016).

Long-term injection of nitrate into the low-temperature (30°C) Medicine Hat Glauconitic C (MHGC) field gave nitrate breakthrough in some producing wells. The oil produced by these wells was found to be depleted in toluene and other alkylbenzenes suggesting that these were favored substrates for hNRB. Alkylbenzene-oxidizing NRB such as *Thauera* and *Azoarcus* were found to be members of the MHGC microbial communities (Agrawal et al., 2012). This suggested that the limited presence of alkylbenzenes prevented complete nitrate reduction.

Light oils may have higher proportions of alkylbenzenes as compared to heavy oils. More (51%) nitrate was reduced with light oil as compared to a heavy oil (15%) in laboratory microcosms. The remaining oils were most depleted in toluene (Lambo et al., 2008). Further incubations of oil field hNRB with 4 mM toluene indicated complete reduction of 8.9 mM nitrate (likely to N_2_) while nitrate was only partially reduced to nitrite in the presence of 4 mM m-xylene. Other alkylbenzenes such as ethylbenzene, propylbenzene, m-ethyltoluene, o-ethyltoluene, or p-ethyltoluene did not support nitrate reduction during the incubation period (Lambo et al., 2008).

Several pure isolates have been reported to use alkylbenzenes for nitrate reduction. Among these, the utilization of toluene by hNRB isolates has been most studied (Spormann and Widdel, 2000; Weelink et al., 2010; Alain et al., 2012). Most of these isolates belong to the *Azoarcus/Thauera* cluster within the class *Betaproteobacteria* (Chakraborty and Coates, 2004). Bacterial strain ToN1, isolated on toluene was found to use crude oil for nitrate reduction. Oil analysis showed utilization of toluene only by this isolate among the oil components (Rabus and Widdel, 1996). Two other strains, mXyN1 and EbN1 isolated on m-xylene and ethylbenzene utilized toluene as well in addition to m-xylene and ethylbenzene, respectively. However, strain PbN1 isolated on n-propylbenzene did not reduce nitrate with crude oil (Rabus and Widdel, 1996; Spormann and Widdel, 2000).

All these studies indicate that not only the availability but also the type and fraction of alkylbenzenes in oil are factors in determining the extent of nitrate reduction by hNRB, which use oil as their substrate. Toluene appears to be a primary electron donor for the reduction of nitrate. The use of oil components to reduce nitrate to N_2_ by hNRB prevents its desired application of sulfide oxidation by soNRB (Voordouw et al., 2009; Agrawal et al., 2012). For successful application of nitrate in limiting sulfide formation in oil reservoirs, it is thus very important to understand the effect of oil composition on nitrate reduction, which is the topic of the current contribution.

## Materials and Methods

### Samples of Oil and Produced Water

Oils are classified as light, light/heavy or heavy depending on their American Petroleum Institute (API) gravity (American Petroleum Institute [API], 2011). These are in excess of 31°, from 21° to 31° and below 21°, respectively. Three light oils, two light/heavy oils as well as heavy MHGC oil were used (**Table 1**). Produced water from producing well 18PW in the MHGC field collected monthly was used to inoculate all cultures (Voordouw et al., 2009). Freshly collected samples of 18PW were stored in a Coy Anaerobic Hood in an atmosphere of 90% N_2_ (v/v) and 10% CO_2_ (N_2_-CO_2_).

**Table 1:** Physicochemical properties of oil samples used in this study.

Oil	Field information; location	API gravity	Viscosity (cP) at 20°C^∗^	Designation
PNG	Conventional oil field; Papua New Guinea	46°	9.4	Light
CPM	Shale oil field in the Bakken formation; Saskatchewan, Canada	41°	10.8	Light
Tundra	Shale oil field in the Bakken formation; Manitoba, Canada	38°	11.8	Light
Gryphon	Shale oil field; Alberta, Canada	31°	16.6	Light/Heavy
Obigbo	Obigbo field; Nigeria	21°	50.9	Light/Heavy
MHGC	Conventional oil reservoir; Medicine Hat, Alberta, Canada	16°	2471	Heavy

*^∗^Determined at a shear rate of 38/s except for MHGC oil, which was at 0.12/s.*

### Quantification of Alkylbenzenes in Oils by Gas Chromatography-Mass Spectrometry (GC-MS)

Oils (1 ml) were diluted with 9 ml of DCM. A 1 μl volume of the DCM extract was injected with an autoinjector (7683B series, Agilent Technologies, Santa Clara, CA, United States) into a gas chromatograph (7890N series, Agilent) equipped with an HP-1 fused silica capillary column (length 50 m, inner diameter 0.32 mm, film thickness 0.52 μm; J&W Scientific) with helium as the carrier gas and connected to a mass-selective detector (5975C inert XL MSD series, Agilent). Duplicate measurements were done on all the oil samples. Concentrations of toluene, ethylbenzene, o-xylene, and m/p-xylene in the oils were calculated based on the standard line obtained from the peak areas of pure individual standard alkylbenzenes (Sigma–Aldrich) analyzed by GC-MS.

### Enrichment of hNRB in Microcosms

hNRB were enriched in 125 ml serum bottles, containing 44 ml of anoxic CSBK medium (Supplementary Table S1) amended with 0, 10, 20, 40, or 80 mM nitrate. To these 1 ml of the oils (2% of total volume of 50 ml), listed in **Table 1**, was added as electron donor and 5 ml of produced water from 18PW was used to inoculate the bottles. Bottles without hNRB inoculum were also prepared. The bottles had a headspace of N_2_-CO_2_, were sealed with sterile butyl rubber stoppers and crimped with aluminum seals. Duplicate serum bottles for all oils and all nitrate concentrations were incubated upside down at 30°C for 90 days, while shaking at 80 rpm.

To a second set of microcosms, additional toluene (71.2–570 mM) was added to 1 ml of oil phase in bottles that showed less than 5 mM reduction of nitrate after 90 days of incubation. These bottles were again incubated upside down at 30°C, while shaking at 80 rpm for an additional 85–90 days.

### Enrichment of hNRB in Hungate Tubes

hNRB were also cultivated in 20 ml Hungate tubes, containing 12.5 ml of anoxic CSBK medium amended with 0, 10, 20, 40, or 80 mM nitrate. The tubes were inoculated with 1.5 ml of produced water from 18PW. As an electron donor, 1 ml of oils (**Table 1**; 6.7% of the total volume of 15 ml) was added to the tubes. Uninoculated tubes without nitrate were also prepared. Tubes were sealed with sterile butyl rubber stoppers, crimped with aluminum seals and contained a headspace of N_2_-CO_2_. Duplicates tubes were incubated upside down at 30°C while shaking at 80 rpm for 90 days.

### Determination of Nitrate Utilization in hNRB Enrichments

The reduction of nitrate in microcosms was monitored as a function of time in serum bottles while nitrate was only measured at the end of incubation period in the Hungate tubes. Samples (200 μl) were transferred to 1.5 ml microfuge tubes using N_2_-CO_2_ flushed syringes and centrifuged at 14,000 rpm for 5 min throughout. Nitrate and nitrite concentrations were measured in the supernatant by High Performance Liquid Chromatography (HPLC) using a UV detector (Gilson, Lewis Center, OH, United States) and an IC-PAK anion column (4 mm × 150 mm, Waters) with borate/gluconate buffer at a flow rate of 2 ml/min (Agrawal et al., 2012). The reduction of nitrate (%) was calculated as: reduction (%) = (nitrite concentration/initial nitrate concentration)^∗^40% + (initial nitrate concentration - residual nitrate concentration - nitrite concentration)/(initial nitrate concentration)^∗^100%.

### Determination of Alkylbenzene Utilization in hNRB Enrichments

To the first set of microcosms a known volume of mesitylene (1,3,5-trimethylbenzene) was added to the oil layer at the end of incubation period. A known volume of dichloromethane (DCM) was added to extract the oils. The oil-DCM layers settled at the bottom of the bottles were carefully taken into 2 ml GC-MS vials and were analyzed using GC-MS as described before. Utilization of a particular alkylbenzene for nitrate reduction was determined as the decrease in its peak area relative to that of mesitylene.

### Biomass Formation in hNRB Enrichments

The contents of Hungate tubes were transferred to 15 ml Falcon tubes at the end of the incubation period and centrifuged at 12,000 rpm for 30 min at 4°C. Supernatants were separated and the cell pellets obtained were washed with 50 mM phosphate buffer (pH 7) twice and re-suspended in 14 ml of the same buffer. Biomass concentrations were determined by measuring the optical density of cell suspensions at 600 nm (OD_600_) using phosphate buffer as a blank. Cell suspensions (100 μl) were also taken in 1.5 ml microfuge tubes, centrifuged at 17,000 × *g* for 5 min followed by re-suspension in 100 μl of 0.1 N NaOH and then mixed well by pipetting up and down. The tubes were incubated in a heating block at 90°C for 10 min and centrifuged again at 15,000 × *g* for 1 min. Protein concentrations were then measured in the supernatant with a Qubit fluorometer (Invitrogen) using a Qubit^®^ protein assay kit (Life Technologies, United States).

### Microbial Community Analysis of hNRB Enrichments

hNRB enrichments from microcosms with oils and the oils with added toluene were centrifuged at 12,000 rpm for 30 min to collect cell pellets at the end of incubation period of 171–180 days. Cell pellets from duplicate incubations were combined before DNA extraction. DNA was extracted using the Fast DNA^®^ Spin Kit for Soil (MP Biomedicals) as per the manufacturer’s instructions and quantified with a Qubit fluorometer (Invitrogen), using the Quant-iT^TM^ dsDNA HS Assay Kit (Invitrogen). 16S rRNA genes of the extracted DNA were amplified using a two-step PCR procedure with each reaction of 50 μl volume containing premade reagents mixed in proportion as per manufacturer’s instructions (Thermo-Scientific). The first PCR used 16S primers 926Fi5 (50-TCGTCGGCAGCGTCAGATGTGTATAAGAGACAGAAACTYAAAKGAATWGRCGG-30) and 1392Ri7 (50-GTCTCGTGGGCTCGGAGATGTGTATAAGAGACAGACGGGCGGTGWGTRC-30) as indicated elsewhere (Menon and Voordouw, 2016). The PCR was for 3 min at 95°C, followed by 25 cycles of 30 s at 95°C, 45 s at 55°C and 90 s incubation at 72°C, followed by a final step at 72°C for 10 min. The quality of the PCR product was evaluated by electrophoresis on a 1.5% (w/v) agarose gel. The PCR product obtained was purified using QIAquick PCR purification kit (Qiagen, Germany) and quantified. This was then used as the template for the second PCR reaction, which used primer P5-S50X-OHAF and P7-N7XX-OHAR for 10 cycles as described elsewhere (Menon and Voordouw, 2016). The resulting purified PCR product was sequenced using the 300PE (paired-end) MiSeq protocol on an Illumina Miseq system at the Department of Geosciences, University of Calgary. The 300PE reads were merged into single reads using PEAR 0.9.6 with a 50 bp overlap and were further processed with a 420 bp cut-off of amplicon size using MetaAmp, a 16S rRNA data analysis pipeline, developed by the Energy Bioengineering Group, Department of Geosciences, University of Calgary. MetaAmp was also used for bioinformatic analysis (http://ebg.ucalgary.ca/metaamp). Raw read sequences have been submitted to NCBI Sequence Read Archive (SRA) under Bioproject accession number PRJNA181037, with biosample number SAMN06624307.

## Results

### Physicochemical Properties and Concentrations of Alkylbenzenes

**FIGURE 1:**
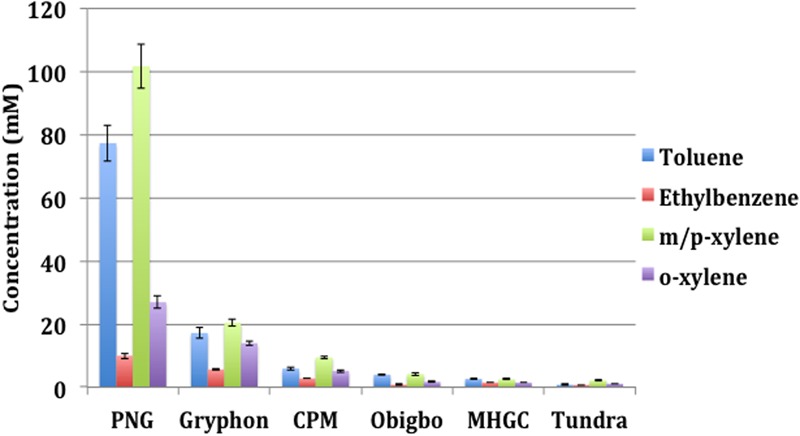
Concentrations of alkylbenzenes determined by gas chromatography-mass spectrometry (GC-MS) analysis of oil samples used in this study. Error bars represent standard deviations for duplicate measurements.

The physicochemical properties of the oils are listed in **Table 1**. PNG, CPM, and Tundra oils were light with API gravities from 38° to 46° and viscosities from 9.4 to 11.8 cP. The Gryphon and Obigbo oils were light/heavy with API gravities of 31° and 21° and viscosities of 16.6 and 50.9 cP, respectively, whereas the MHGC oil was heavy with an API gravity of 16° and a viscosity of 2471 cP. The concentrations of the alkylbenzenes toluene, ethylbenzene, o-xylene, and m/p-xylene differed for these oils (**Figure 1**). Of the light oils PNG oil had the highest concentrations of alkylbenzenes, whereas those of CPM oil and Tundra oil were much lower (**Figure 1**). The latter two are shale oils from the Bakken formation (**Table 1**), which have a low fraction of aromatics (Jiang and Li, 2002). Of the light/heavy oils Gryphon oil had a higher content of alkylbenzenes than Obigbo oil, whereas the alkylbenzene content of heavy MHGC oil was intermediate between that of Obigbo and Tundra oils (**Figure 1**). The toluene concentrations of PNG, CPM, Tundra, Gryphon, Obigbo, and MHGC oils were found to be 77, 5.9, 0.8, 17, 4.0, and 2.6 mM, respectively (**Figure 1**). Assuming toluene to be the most important substrate for nitrate reduction (Rabus and Widdel, 1996; Lambo et al., 2008; Agrawal et al., 2012) we expect nitrate reduction to be ranked as follows: PNG > Gryphon > CPM > Obigbo > MHGC > Tundra.

### Nitrate Reduction Using Oils as Electron Donors

**FIGURE 2:**
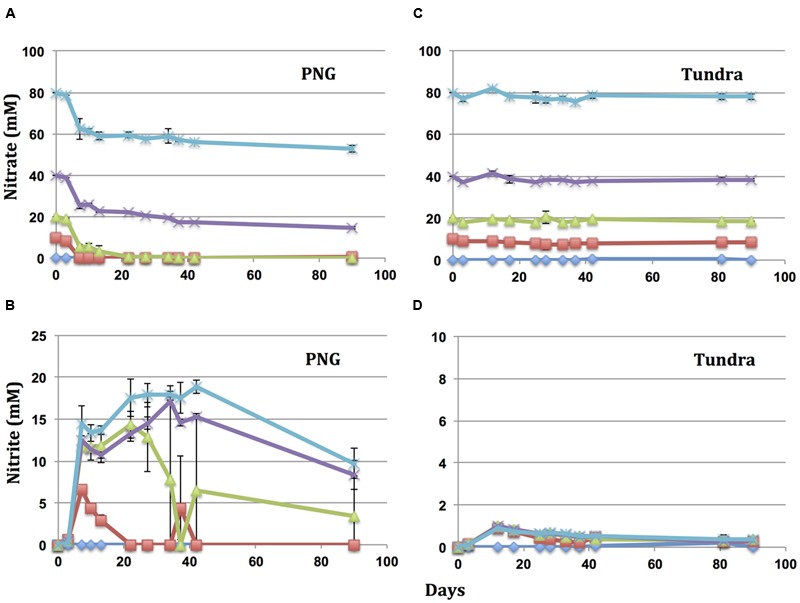
Reduction of nitrate and formation of nitrite as a function of time in microcosms with PNG oil **(A,B)** and Tundra oil **(C,D)**. All microcosms were inoculated with a 10% (v/v) inoculum of produced water from 18PW in the MHGC field. The initial nitrate concentrations were 80 (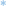), 40 (×), 20 (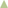), 10 (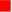), or 0 (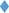) mM. Error bars represent the standard deviations for four measurements.

Oils were incubated in microcosms with different concentrations of nitrate and the same inoculum from producing well 18PW in the MHGC field. Incubation of PNG oil with 80, 40, 20, and 10 mM nitrate gave reduction of 23.0 ± 2.4, 21.5 ± 2.8, 19.6 ± 0.4, and 10.0 ± 0.4 mM nitrate, respectively (**Figure 2A**). Hence, with nitrate in excess, the maximum concentration reduced was 22.2 ± 1.1 mM (**Table 2**). Reduction of nitrate led to transient formation of nitrite with maximum concentrations of 18.0 ± 0.5, 14.9 ± 1.4, 11.7 ± 2.1, and 6.6 ± 0.03 mM nitrite, respectively, and final concentrations of 9.7 ± 1.8, 8.3 ± 1.7, 3.3 ± 4.7, and 0 mM, respectively (**Figure 2B**). Incubation with Tundra oil gave reduction of only 1.4 ± 0.6 mM of nitrate (i.e., nitrate was in excess in all four incubations) with formation of maximally 0.4 ± 0.1 mM nitrite (**Figures 2C,D** and **Table 2**). Likewise incubations with Gryphon, CPM, Obigbo, and MHGC oils gave maximal reduction of 12.3 ± 1.6, 7.9 ± 0.6, 4.6 ± 0.7, and 4.0 ± 1.5 mM nitrate (**Table 2**) with transient formation of up to 8.7 ± 0.4, 3.9 ± 1.1, 2.1 ± 0.3, and 1.6 ± 0.3 mM nitrite (Supplementary Figure S1). Overall, we observed that nitrate reduction followed the order PNG > Gryphon > CPM > Obigbo > MHGC > Tundra, which was the same as that based on toluene concentrations in the oils.

**Table 2:** Maximum concentrations of nitrate reduced and nitrite produced at the end of incubations in serum bottles with 2% (v/v) of oil and in Hungate tubes with 6.7% (v/v) of oil.

Oil	2% (v/v) of oil				6.7% (v/v) of oil				Fold increase in nitrate reduced
	*N*	Nitrate reduced (mM)	Nitrite formed (mM)	Theoretical maximum toluene contribution (%)	N	Nitrate reduced (mM)	Nitrite formed (mM)	Theoretical maximum toluene contribution (%)	
PNG	6	22.2 ± 1.1	9.5 ± 7.3	68	2	80.0 ± 0ˆ*	39.7 ± 3.2	66	3.6
Gryphon	6	12.3 ± 1.6	3.9 ± 2.7	25	4	41.7 ± 5.2	32.3 ± 6.4	37	3.4
CPM	8	7.9 ± 0.6	2.6 ± 0.7	13	4	27.7 ± 0.8	23.2 ± 2.2	21	3.5
Obigbo	8	4.6 ± 0.7	1.4 ± 0.2	15	6	16.2 ± 3.4	14.8 ± 1.9	26	3.2
MHGC	8	4.0 ± 1.5	0.7 ± 0.2	11	6	12.5 ± 2.1	8.7 ± 1.3	17	2.2
Tundra	8	1.4 ± 0.6	0.4 ± 0.1	10	8	6.9 ± 3.2	1.2 ± 1.1	6	4.9

*^∗^ No nitrate remained in this incubation.*

*Toluene contribution (%) = V_*oil*_/V_*aq*_ [(5^∗^((C_*nitrate reduced*_ - C_*nitrite*_) + 2^∗^ C_*nitrite*_))/36] ^∗^ 100, where 36 is the number of electrons released for oxidation of toluene to 7CO_2_.*

*The average ± SD is for incubations with 10, 20, 40, or 80 mM nitrate in which some nitrate remained. The number of replicates (*N*) to which this applies is indicated.*

Increasing the volume fraction of oil from 1 ml per 50 ml (2% v/v) in serum bottles to 1 ml per 15 ml (6.7% v/v) in Hungate tubes increased the reduction of nitrate to 80, 41.7 ± 5.2, 27.7 ± 0.8, 16.2 ± 3.4, 12.5 ± 2.1, and 6.9 ± 3.2 mM with PNG, Gryphon, CPM, Obigbo, MHGC, and Tundra oil, respectively (**Table 2** and Supplementary Figure S2). Hence, increasing the volume fraction of oils used as electron donor by 3.3-fold increased the reduction of nitrate on average by 3.5-fold (**Table 2**).

**FIGURE 3:**
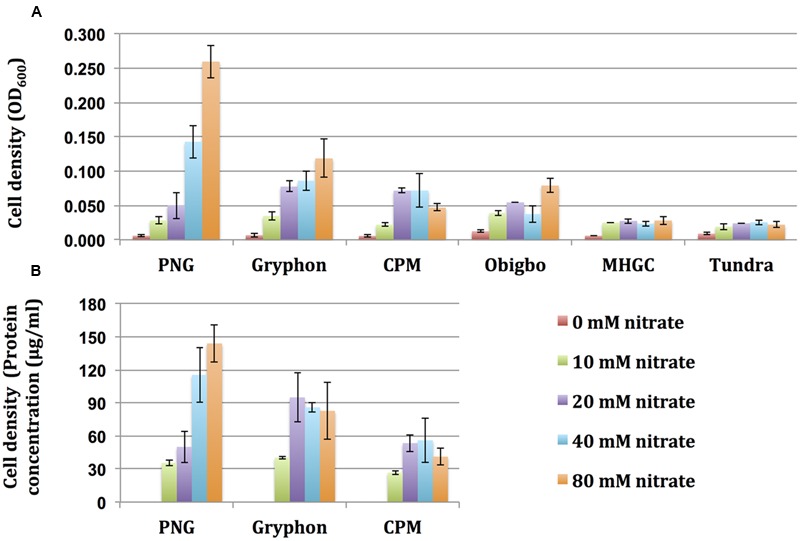
Biomass concentration as a function of nitrate reduction in Hungate tube incubations with oils as electron donors. Cell density is represented by **(A)** Optical density measured at 600 nm and **(B)** Protein concentration (μg/ml). Error bars represent standard deviations of duplicate measurements.

The maximum concentration of nitrate reduced with different oils as a function of their toluene concentrations is indicated in **Figure 3**. The maximum contribution which toluene in the oil can make to this reduction is also indicated in **Table 2**. These data indicate that components other than toluene also contributed to the observed reduction of nitrate. This contribution was least for high toluene PNG oil (32–34%) and most for low toluene Tundra oil (90–94%). The fraction of nitrate reduction to nitrite was on average 31% in serum bottles and 65% in Hungate tubes. Hence, increasing the ratio of oil to nitrate increased the concentration of nitrate reduced to nitrite, but nitrate reduction was less complete.

### Biomass Concentration as a Function of Nitrate Reduction

**FIGURE 4:**
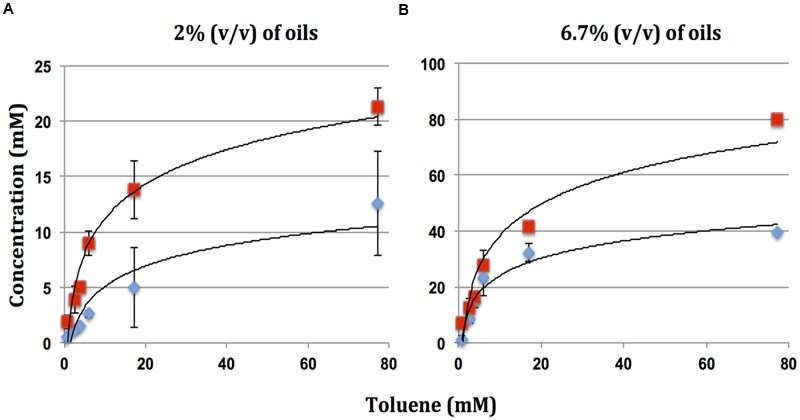
Maximum reduction of nitrate (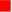) and formation of nitrite (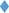) in microcosms incubated with oils with different toluene concentrations for 90 days. Data are for **(A)** serum bottles and **(B)** Hungate tubes. Error bars represent the standard deviations for eight replicate measurements.

The concentration of biomass formed in the Hungate tubes was measured as the OD_600_ (turbidity) of the aqueous phase or as the protein concentration of the aqueous phase, which showed similar trends (**Figure 4**). The biomass concentration increased with increased reduction of nitrate and was, therefore highest with PNG oil and lowest with Tundra oil (**Figure 4** and **Table 2**). These observations indicate that increased nitrate reduction coupled to oil organics oxidation increased biomass formation.

### Selective Utilization of Oil Components for Nitrate Reduction

**FIGURE 5:**
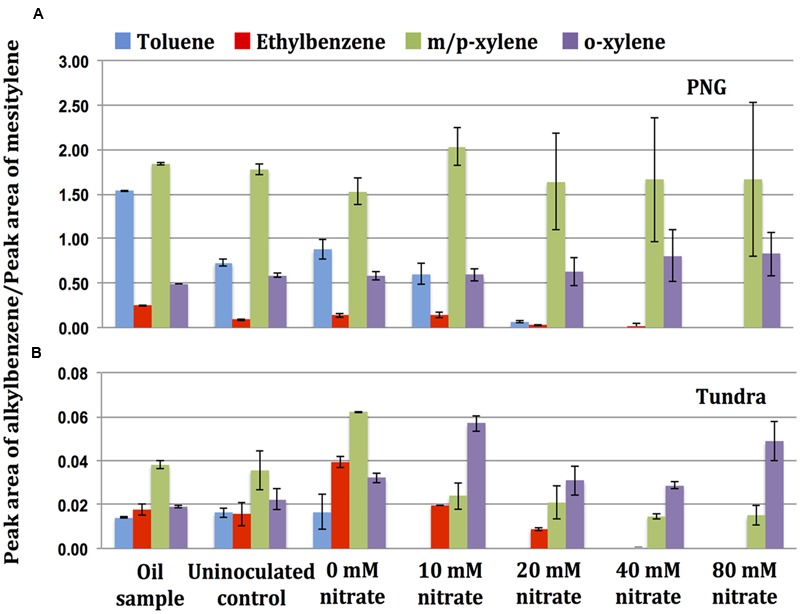
Gas chromatography-mass spectrometry analysis of the oils, **(A)** PNG and **(B)** Tundra extracted from the incubations of oil field hNRB with nitrate and oils at the end of incubation period of 90 days. The presence of alkylbenzenes is represented as the ratio of peak areas of a particular alkylbenzene to mesitylene (1,3,5-trimethylbenzene). Note the difference in scale. Error bars represent standard deviations for duplicate measurements.

Gas chromatography-mass spectrometry analysis of the oils incubated with nitrate and 18PW inoculum indicated use of alkylbenzenes for nitrate reduction (**Figure 5** and Supplementary Figure S3). Results showed that hNRB primarily used toluene as an electron donor for nitrate reduction. As compared to uninoculated enrichments without nitrate, toluene was completely utilized (0–1% remaining) in incubations of oils with 80 mM nitrate (**Figure 5** and Supplementary Figure S3). Oils incubated with lower concentrations of nitrate (10, 20, or 40 mM) were also depleted in toluene. Toluene was completely removed from oils with the lowest concentrations of toluene (e.g., from Tundra oil) even in incubations with 10 mM nitrate (**Figure 5** and Supplementary Figure S3).

Nitrate reduction was coupled to the utilization of other alkylbenzenes, including ethylbenzene and m/p-xylene, but not o-xylene. Oils extracted from serum bottles with 80 mM nitrate had 0–5% residual ethylbenzene and 25–93% of m/p-xylene at the end of the incubation period (**Figure 5** and Supplementary Figure S3). Hence, the use of alkylbenzenes may account for all of the nitrate reduction for PNG oil. However, nitrate reduction with the other oils must have involved oxidation of other oil components. This was clearly a slower process causing most nitrate to remain.

### Increased Nitrate Reduction by Toluene Addition

**FIGURE 6:**
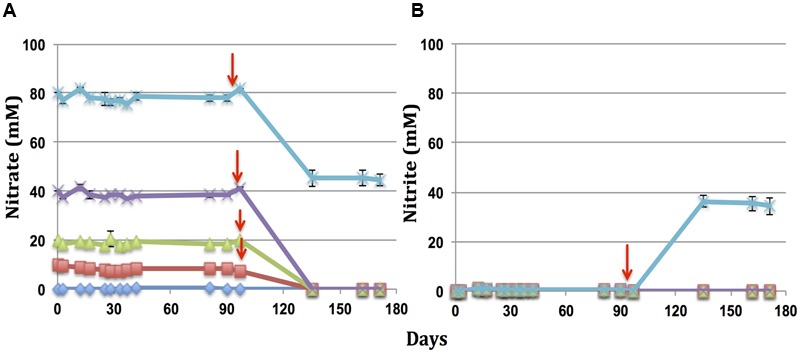
Reduction of nitrate **(A)** and formation of nitrite **(B)** as a function of time in microcosms with Tundra oil amended with toluene at the indicated time (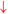). Incubations contained 49 ml of inoculated medium with 80 (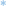), 40 (×), 20 (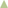), 10 (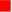), or 0 (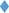) mM nitrate and 1 ml of Tundra oil. The oil phase was amended with additional 570, 285, 142, and 71 mM of toluene, respectively. Error bars represent the standard deviations for duplicate measurements.

The GC-MS analysis suggested that hNRB preferentially used toluene for nitrate reduction. Less nitrate was reduced in incubations of oils with less toluene. We tested, therefore, whether toluene addition to low toluene oils increased nitrate reduction. This was evaluated by adding 71, 142, 285, or 570 mM of toluene to 1 ml of oil in serum bottles with a 50 ml volume containing 10, 20, 40, or 80 mM nitrate, respectively, at day 90 of incubation (**Figure 6**). After 45 days of further incubation, microcosms containing toluene-amended Tundra oil and 10, 20, or 40 mM of nitrate showed complete reduction of nitrate without accumulation of nitrite indicating complete reduction of nitrate to N_2_ (**Figure 6**). The microcosm with 570 mM toluene in the oil phase and 80 mM nitrate in the aqueous medium showed reduction of 32.3 ± 1.3 mM nitrate to nitrite, which was not reduced further even after continued incubation for an additional 36 days (**Figure 6** and Supplementary Figure S4). Similar results were obtained in incubations with other oils. Incubations with 10, 20, or 40 mM nitrate and oils with 71, 142, or 285 mM toluene, respectively, showed mostly complete reduction of nitrate (results not shown). Incubation of other oils with 570 mM of additional toluene in the oil phase and 80 mM nitrate in the aqueous phase gave on average reduction of 34.8 ± 4.2 mM nitrate (*N* = 12) with accumulation of 30.7 ± 4.7 mM nitrite (*N* = 12; Supplementary Figure S4). These observations confirmed that remediating toluene deficiency of an oil increases nitrate reduction, irrespective of whether the oil is light, light/heavy, or heavy.

### Microbial Community Compositions of hNRB Enrichments

**FIGURE 7:**
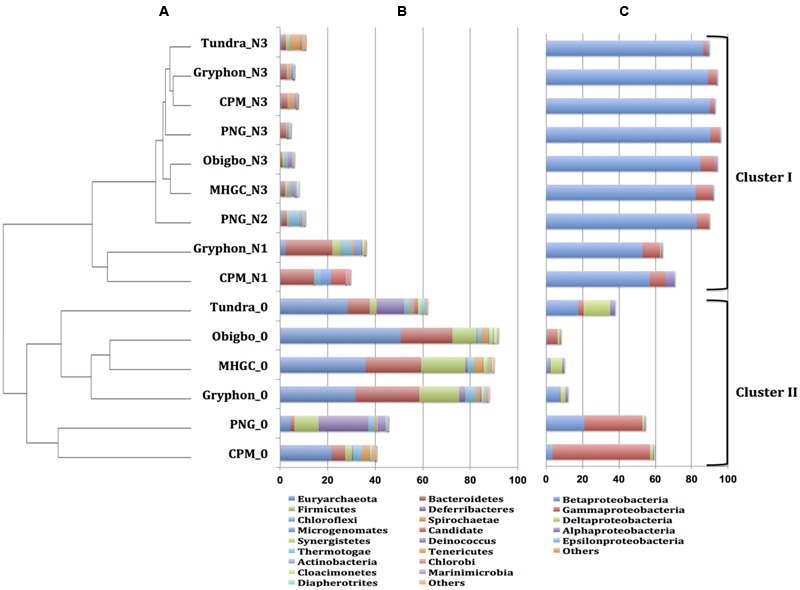
Comparison of microbial community compositions derived from 16S rRNA amplicons of hNRB incubations with oils. **(A)** Relational tree generated using the UPGMA algorithm in MetaAmp. Microbial community compositions are represented at **(B)** at the phylum level excluding *Proteobacteria* and **(C)** at the class level for *Proteobacteria* only. Data are for incubations with the indicated oils without nitrate (0), with 10 mM nitrate (N1), with 10 and 20 mM nitrate (N2) or for incubations with additional toluene and 10, 20, 40, and 80 mM nitrate (N3), as in **Figure 5**; chromosomal DNA’s for these latter incubations with a given oil were combined.

Microbial community compositions derived from 16S rRNA amplicons from the hNRB incubations with oils are compared in **Figure 7**. The dendrogram showed two main clusters I and II, representing incubations with and without nitrate, respectively (**Figure 7A**). The phylum *Proteobacteria*, class *Betaproteobacteria* dominated the community compositions in cluster I, whereas the phylum *Euryarchaeota* dominated the community compositions in cluster II (**Figures 7B,C**). Microbial community compositions in cluster I were dominated by *Betaproteobacteria* of the genus *Thauera* especially for incubations with nitrate and added toluene in which the communities consisted of 80–88% of this taxon (**Table 3**). *Thauera* is a known denitrifying hNRB, which oxidizes toluene (Spormann and Widdel, 2000; Chakraborty and Coates, 2004; Hubert and Voordouw, 2007; Fida et al., 2016). *Pseudomonas*, an extensively studied hNRB of the class *Gammaproteobacteria* (Altenschmidt and Fuchs, 1991; Grigoryan et al., 2008), was the second most dominant genus in this cluster. The proportions of *Thauera* were lower in cluster II (0.6–21%). Cluster II had high fractions of methanogens such as *Methanosaeta* (0.03–28%), *Methanocalculus* (3–7%), *Methanoculleus* (0.4–6%), and *Methanofollis* (0.2–2%). In cluster I, the fractions of these methanogens were less than 1.5% (**Table 3**).

**Table 3:** Microbial community compositions of hNRB enrichments.

		Cluster I									Cluster II					
#Taxonomy (Class; Order; Family; Genus)	Average	Tundra_N3	Gryphon_N3	CPM_N3	PNG_N3	Obigbo_N3	MHGC_N3	PNG_N2	Gryphon_N1	CPM_N1	Tundra_0	Obigbo_0	MHGC_0	Gryphon_0	PNG_0	CPM_0
Betaproteobacteria; Rhodocyclales; Rhodocyclaceae; Thauera	**48.5**	**84.2**	**85.0**	**88.2**	**83.7**	**82.7**	**80.1**	**75.7**	**45.2**	**51.2**	**17.0**	**0.6**	**1.9**	**7.5**	**20.5**	**3.5**
Gammaproteobacteria; Pseudomonadales; Pseudomonadaceae; Pseudomonas	**9.5**	**1.7**	**4.4**	**1.9**	**5.0**	**7.3**	**9.1**	**6.3**	**9.5**	**5.9**	**2.7**	**3.4**	0.4	0.3	**31.6**	**53.3**
Sphingobacteriia; Sphingobacteriales; WCHB1-69	**6.9**	0.7	**1.8**	**1.2**	0.5	0.6	**1.1**	0.4	**15.7**	**10.8**	**5.2**	**20.2**	**18.9**	**23.6**	0.3	**2.6**
Methanomicrobia; Methanosarcinales; Methanosaetaceae; Methanosaeta	**6.6**	0.0	0.0	0.0	0.0	0.0	0.0	0.0	0.1	0.0	**12.1**	**28.4**	**25.9**	**18.1**	0.0	**14.5**
Methanomicrobia; Methanomicrobiales; Methanomicrobiales-Incertae-Sedis; Methanocalculus	**2.6**	0.0	0.0	0.0	0.0	0.0	0.0	0.4	**1.3**	0.0	**12.8**	**6.2**	**4.3**	**6.9**	**3.4**	**3.8**
Deferribacteres; Deferribacterales; Deferribacteraceae	**2.5**	0.0	0.0	0.0	0.0	0.0	0.0	0.0	0.1	0.0	**11.5**	0.3	0.9	**2.6**	**20.8**	0.5
Bacteroidia; Bacteroidales; Porphyromonadaceae; Proteiniphilum	**1.8**	0.7	0.4	**1.2**	**1.7**	0.1	0.2	**1.4**	**3.1**	**3.2**	**3.5**	**1.2**	**3.0**	**2.9**	**1.2**	**3.0**
Anaerolineae; Anaerolineales; Anaerolineaceae	**1.7**	0.3	0.2	0.1	0.0	**1.3**	0.6	**3.3**	**3.5**	**2.6**	**2.0**	**1.3**	**2.3**	**3.2**	**1.9**	**2.8**
Clostridia; Clostridiales; Eubacteriaceae; Acetobacterium	**1.4**	0.0	0.0	0.0	0.0	0.0	0.0	0.0	0.0	0.0	0.0	**2.2**	**14.2**	0.0	**4.9**	0.1
Deltaproteobacteria; Syntrophobacterales; Syntrophaceae; Smithella	**1.3**	0.0	0.0	0.0	0.0	0.0	0.0	0.0	0.0	0.0	**13.4**	0.6	**3.8**	**1.8**	0.0	0.5
Methanomicrobia; Methanomicrobiales; Methanomicrobiaceae; Methanoculleus	**1.3**	0.4	0.1	0.5	0.0	0.0	0.2	0.3	0.7	0.1	**2.6**	**5.0**	**1.4**	**5.8**	0.4	**2.3**
Clostridia; Clostridiales; Peptococcaceae	**1.0**	0.0	0.0	0.0	0.0	0.1	0.0	0.0	0.1	0.0	0.8	**1.1**	**2.1**	**11.3**	0.2	0.0
**Total reads**	**414570**	**27365**	**27828**	**19220**	**33375**	**30297**	**29162**	**33228**	**27373**	**31623**	**31623**	**21499**	**28059**	**30608**	**24053**	**24436**

*These are presented in the same order as in the dendrogram (**Figure 7**). Taxa with an average fraction in excess of 1.0% of total Illumina reads are presented in decreasing order. N3, N2, N1 and 0 represent incubations of the indicated oils with 10–80 mM, 10–20 mM, 10 mM nitrate or without nitrate, respectively.*

## Discussion

The use of nitrate to control oil reservoir souring has been studied for the past 20 years (Youssef et al., 2009; Gieg et al., 2011). Its injection in a limited number of wells in the heavy-oil producing, low-temperature Coleville field in Saskatchewan, Canada, decreased sulfide concentrations in produced waters from connected producing wells, decreased numbers of SRB, while increasing those of NRB (Telang et al., 1997). Molecular biology analysis indicated large increases in the fraction of the soNRB strain CVO (Coleville organism), now referred to as *Sulfurimonas* sp. strain CVO, in the microbial community in producing wells. Strain CVO oxidizes sulfide to sulfur or sulfate, while reducing nitrate to nitrite and nitrogen (Gevertz et al., 2000; Lin et al., 2008), explaining the success of this initial field test.

Since then successful control of the oil field sulfur cycle by continuous injection of 50–100 ppm (1–2 mM) of nitrate has been demonstrated, especially in deeper and therefore hotter fields in the North Sea (Sunde and Torsvik, 2005). The temperature limit of life in oil fields has been estimated to be at 80–90°C (Magot, 2005). However, cooling of the near injection wellbore region (NIWR) by injected seawater creates a thermal viability shell in which microbes including SRB and sulfate-reducing *Archaea* can thrive. This region is limited in size with the bulk of the reservoir remaining too hot for microbial activity. Thus control of souring is required in a limited region only, which may explain the reported success of nitrate injection in high-temperature fields (Larsen, 2002; Larsen et al., 2004; Sunde and Torsvik, 2005). However, a more recent evaluation of souring control by nitrate injection in hot North Sea oil fields indicated only partial success over the longer term (Mitchell et al., 2010). Souring stabilized, decreased or kept on increasing gradually in fields injected with nitrate. Interestingly, nitrate reduction in oil fields appears to stop at nitrite at or above 50°C, causing nitrite accumulation and strong inhibition of SRB activity (Greene et al., 2003; Haveman et al., 2004; Fida et al., 2016). Thus souring control with nitrate in high-temperature fields may be improved if the temperature of the NIWR can be prevented from dropping below this temperature.

Monitoring of nitrate injection to control souring in the low-temperature MHGC field has indicated why nitrate injections may fail (Voordouw et al., 2009). Reduction of nitrate to nitrite by hNRB inhibits SRB, but the subsequent reduction of nitrite to N_2_ or ammonium (90 and 10%, respectively; Cornish-Shartau et al., 2010) removes the inhibition. Unfortunately, continuous injection of water containing nitrate and sulfate will cause the reduction of nitrate, followed by reduction of sulfate and then oil-dependent methanogenesis in separate zones along the injection water flow path (Voordouw et al., 2009; Agrawal et al., 2012; Chen et al., 2017). The injected nitrate is reduced to N_2_ by hNRB using oil components as electron donor under these conditions and does not reach the deeper adjacent zone in which sulfate is reduced to sulfide.

If hNRB were to use a wide variety of oil components then these observations would seem to preclude the successful use of nitrate in inhibiting reservoir souring. However, incubations of MHGC oil and produced water with nitrate and/or sulfate indicated that the hNRB were specific in the use of alkylbenzenes with a further preference for toluene (Lambo et al., 2008; Agrawal et al., 2012), whereas SRB used a broader suite of oil components (Agrawal et al., 2012). This hNRB specificity was expressed in the MHGC field by the fact that nitrate breakthrough in a producing well was associated with the production of toluene-free oil (Agrawal et al., 2012). In view of these observations it appears that oil composition, especially toluene content, could be a major determinant of the success of nitrate injections.

Selective use of alkylbenzenes in oil, especially toluene, ethylbenzene, and m-xylene, by strains ToN1, mXyN1, and EnN1 was shown by Rabus and Widdel (1996). Although the phylogeny of these strains was not identified these may have included *Thauera aromatica* and *Azoarcus tolulyticus* strains (Spormann and Widdel, 2000). Reviews have indicated that the majority of hNRB use toluene as electron donor (Spormann and Widdel, 2000; Chakraborty and Coates, 2004); hNRB using benzene are rare (Chakraborty and Coates, 2004). The metabolism of denitrifying *Betaproteobacteria* strain HxN1 using alkanes as electron donor for nitrate reduction has been characterized in detail (Grundmann et al., 2008) and an *Azoarcus* sp., capable of using heptane as electron donor for nitrate reduction has been enriched from the MHGC field (Kryachko and Voordouw, 2014). However, these are not major community components in enrichments with oil and nitrate and nitrate remained in these incubations, despite the presence of significant alkanes in light and heavy oil (Lambo et al., 2008; Agrawal et al., 2012; this study). Incubations of MHGC oil, toluene or VFA and nitrate with MHGC produced waters stimulated the growth of *Thauera* and *Pseudomonas* spp. (Lambo et al., 2008, Voordouw et al., 2009; Gassara et al., 2015; Fida et al., 2016; Chen et al., 2017). These were also found in high proportions in other low-temperature oil fields (Li et al., 2014) and the effect of nitrate to toluene ratio on nitrate reduction by *Pseudomonas* sp. has been studied (Kim et al., 2013). Incubation of MHGC produced water 18PW with toluene-amended oils and nitrate gave very high fractions of *Thauera* (45–88%) and lower fractions of *Pseudomonas* (1.7–9.5%) (**Table 3**).

Note that the high concentrations of nitrate of up to 80 mM which we added to microcosms, greatly exceed the 1–2 mM concentrations typically injected into oil fields to control souring. We added these to determine the maximum concentrations of nitrate that could be reduced per ml of oil. Using this approach, we have found that more nitrate was reduced in incubations of oils with higher concentrations of toluene (e.g., PNG oil), as compared to the oils with lower concentrations of toluene (e.g., Tundra oil) as electron donor (**Figure 1** and **Table 2**). PNG and Tundra are both light oils with a high API gravity and low viscosity (**Table 1**). However, the lightness of the Tundra oil is caused by high concentrations (9 mM) of low molecular weight alkanes, like pentane and heptane (Jiang and Li, 2002; Menon and Voordouw, 2016). Its toluene content is very low (**Figure 1**), as has been observed for many other shale oils (Jiang and Li, 2002). Thus, despite the presence of a heptane-utilizing *Azoarcus* sp. in MHGC produced water (Kryachko and Voordouw, 2014) and the presence of low molecular weight alkanes in Tundra oil (Menon and Voordouw, 2016), nitrate reduction was limited by its low toluene content. Amendment of Tundra oil with additional toluene increased the reduction of nitrate (**Figure 6A**) through the activity of *Thauera*, which increased from 17 to 84% (**Table 3**). Toluene limitation would not affect the reduction of sulfate to sulfide, because SRB use a much wider range of oil components, including alkanes, as substrates (Davidova et al., 2006; Savage et al., 2010; Agrawal et al., 2012; Sherry et al., 2013).

We may, therefore, expect that when nitrate is injected into a reservoir with low toluene oil such as Tundra, it will penetrate deeper and will be more available to soNRB like *Sulfurimonas* for oxidizing SRB-produced sulfide than when it is injected in a high-toluene reservoir such as PNG. When injecting nitrate in high-toluene reservoir such as PNG, we expect extensive nitrate reduction and formation of high hNRB biomass concentrations (**Table 2** and **Figure 3**). This biomass may be surface-active itself or may produce biosurfactant causing oil emulsification increasing oil recovery (Youssef et al., 2009; Kryachko and Voordouw, 2014; Li et al., 2014; Gassara et al., 2017). Increasing the toluene-content of oil can be done by injecting water-dissolved toluene, which transfers to the oil phase. Subsequent injection of water-dissolved nitrate gives toluene and nitrate-mediated microbially enhanced oil recovery (MEOR; Gassara et al., 2015).

Thus, the toluene content of the oil in a reservoir is an important determinant of the success of nitrate injection for souring control and MEOR. If the goal is to control souring, then the toluene content should be low. If the goal is MEOR, then it should be high either naturally or through injection.

## Author Contributions

NS: Experiments, data collection, data analysis and interpretation, drafting the manuscript, critical revision of the manuscript. JV: DNA work. GV: Idea of the work, supervision, final approval of the manuscript to be published.

## Conflict of Interest Statement

The authors declare that the research was conducted in the absence of any commercial or financial relationships that could be construed as a potential conflict of interest.
