# Supplementary material for: The Effectiveness of Nitrate-Mediated Control of the Oil Field Sulfur Cycle Depends on the Toluene Content of the Oil
Source: Front Microbiol. 2017 May 31;8:956. doi: 10.3389/fmicb.2017.00956 (PMC5450463; doi:10.3389/fmicb.2017.00956)
Supplement: Supplementary file 1 [file Data_Sheet_1.PDF]

Supplementary Material

The effectiveness of nitrate-mediated control of the oil field sulfur cycle depends on the toluene content of the oil

Navreet Suri, Johanna Voordouw and Gerrit Voordouw*

Petroleum Microbiology Research Group, Department of Biological Sciences, University of Calgary, Calgary, AB, Canada

*** Correspondence:** Gerrit Voordouw, Petroleum Microbiology Research Group, Department of Biological Sciences, University of Calgary, 2500 University Drive NW, Calgary, AB, T2N 1N4, Canada.

voordouw@ucalgary.ca

1 Supplementary Figures and Tables

1.1 Supplementary Tables

Table S1 | Components of CSBK medium per L

• 1.5 g NaCl, 0.05 g KH_2PO_4, 0.32 g NH_4Cl, 0.21 g $CaCl_2 \cdot 2H_2O$, 0.54 g $MgCl_2 \cdot 5H_2O$, 0.1 g KCl and 30 ml of 1 M sodium bicarbonate, pH 7
• 1 ml of trace elements (Widdel and Bak, 1992); see below
• 1 ml of tungstate and selenite (Widdel and Bak, 1992); see below
• Trace elements: 993 ml of deionized water, 6.5 ml of HCl (25%, w/v), 1.5 g of $FeCl_2 \cdot 4H_2O$, 60 mg H_3BO_3, 100 mg $MnCl_2 \cdot 4H_2O$, 120 mg $CoCl_2$ $6H_2O$, 70 mg $ZnCl_2$, 25 mg $NiCl_2 \cdot 6H_2O$, 15 mg $CuCl_2 \cdot 2H_2O$ and 25 mg $Na_2MoO_4 \cdot 2H_2O$.
• Tungstate and selenite: 1000 ml of deionized water, 8 mg of Na_2WO_4, 6 mg of $Na_2SeO_3 \cdot 5H_2O$, 400 mg NaOH

1.2 Supplementary Figures

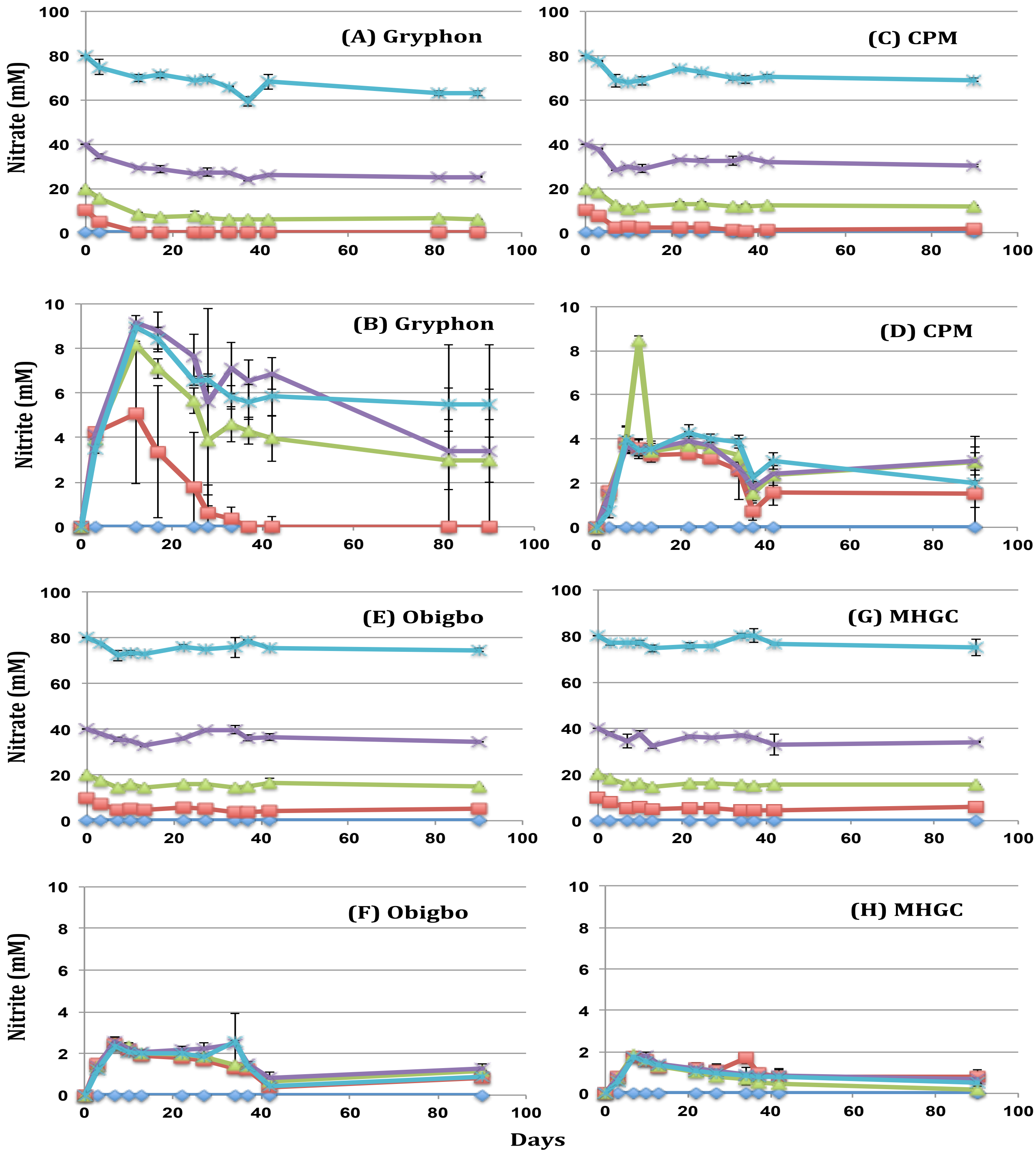

Figure S1 | Reduction of nitrate and formation of nitrite as a function of time in microcosms with Gryphon oil (A, B), CPM oil (C, D), Obigbo oil (E, F) and MHGC oil (G, H). All microcosms were inoculated with a 10% (v/v) inoculum of produced water from 18PW in the MHGC field. The initial nitrate concentrations were 80 (✳), 40 (✕), 20 (▲), 10 (■) or 0 (◆) mM. Error bars represent the standard deviations for four measurements.

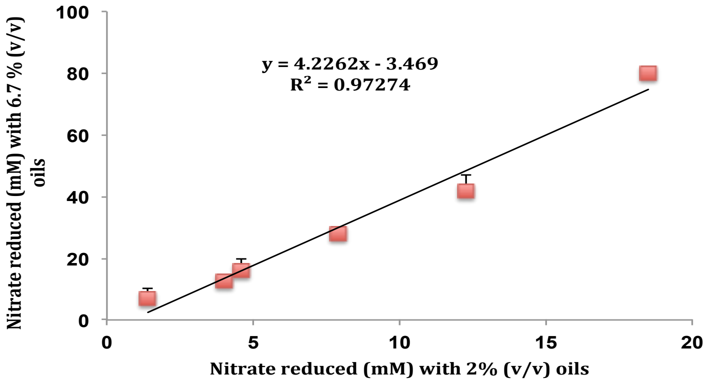

Figure S2 | Increase in nitrate reduction with the increased volume fraction of oils as electron donors. Each point is an average ± SD of 8 replicates.

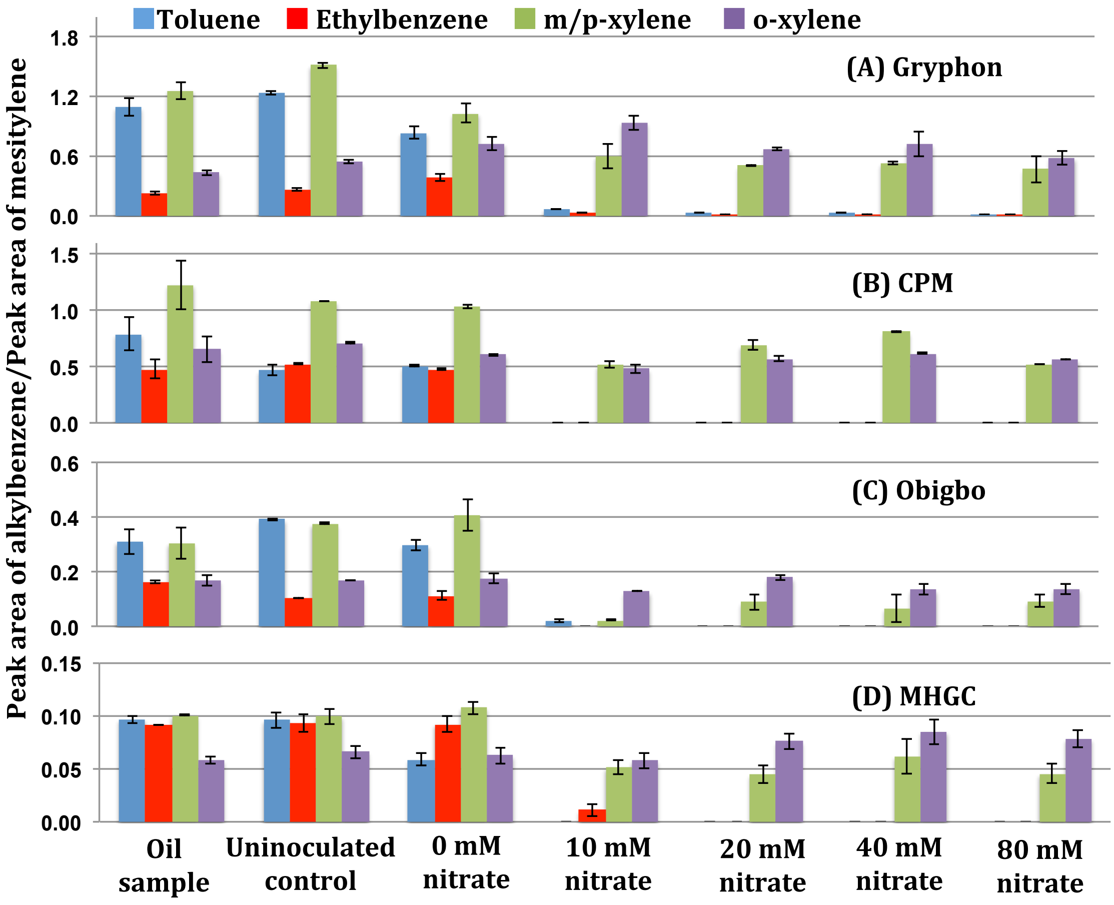

Figure S3 | GC-MS analysis of the oils, (A) Gryphon, (B) CPM, (C) Obigbo and (D) MHGC extracted from the incubations of oil field hNRB with nitrate and oils at the end of incubation period of 90 days. The presence of alkylbenzenes is represented as the ratio of peak areas of a particular alkylbenzene to mesitylene (1,3,5-trimethylbenzene). Note the difference in scale. Error bars represent standard deviations for duplicate measurements.

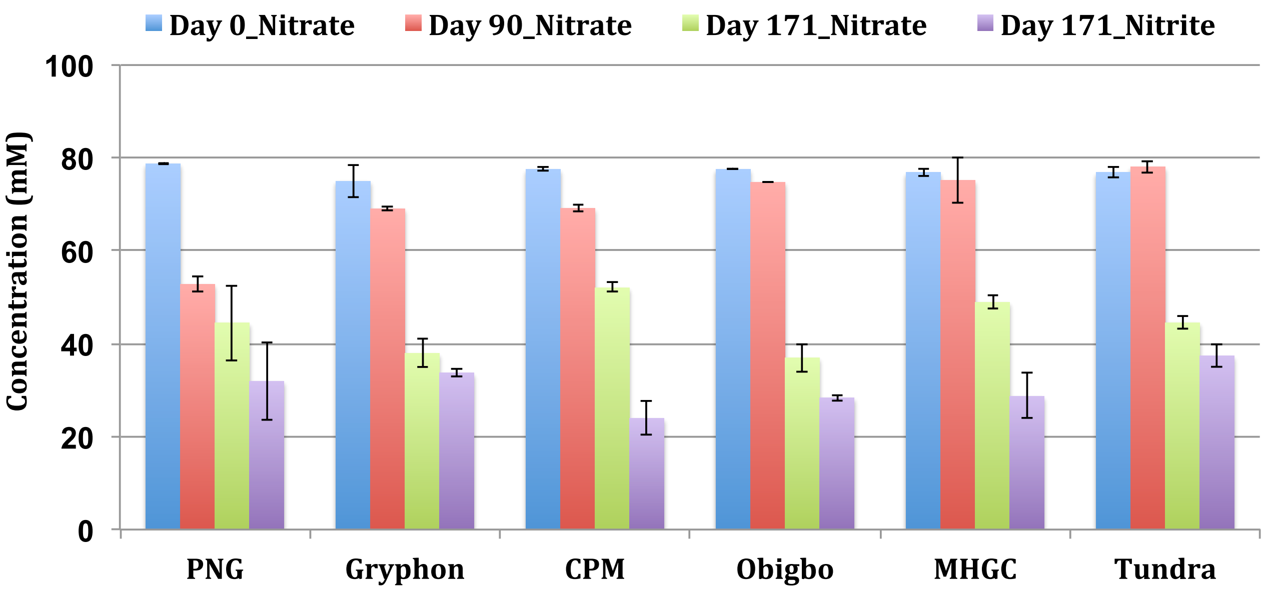

Figure S4 | Reduction of nitrate in 49 ml of microcosms with 80 mM nitrate and 1 ml of different oils as indicated. An additional 570 mM toluene was added after 90 days of incubation, as in Figure 6. Incubation was then continued and the residual concentrations of nitrate and of nitrite produced are shown. Error bars represent standard deviations for duplicate measurements.
